# Integrated Phenotypic and Genomic Analysis of Antimicrobial Resistance, Virulence, and Phylogeny in *Vibrio cholerae* Isolates from Jiaxing, China, with Emphasis on Non-O1/Non-O139 Strains

**DOI:** 10.3390/microorganisms14040813

**Published:** 2026-04-02

**Authors:** Miaomiao Jia, Ping Li, Zhongwen Chen, Xuejuan Liu, Lei Gao, Guoying Zhu, Yong Yan

**Affiliations:** Jiaxing Key Laboratory of Pathogenic Microbiology, Jiaxing Center for Disease Control and Prevention, Jiaxing 314050, China; jmm0649@163.com (M.J.); plijxcdc@163.com (P.L.); czw2007@sohu.com (Z.C.); liuxuejuan@foxmail.com (X.L.); shuangyuzuo007@126.com (L.G.); jxcdczhuguoying@163.com (G.Z.)

**Keywords:** aquatic surveillance, genomic epidemiology, cgMLST, quinolone resistance, environmental reservoirs

## Abstract

Non-O1/non-O139 *Vibrio cholerae* strains are widely distributed in aquatic environments worldwide and are increasingly recognized as potential reservoirs of antimicrobial resistance and virulence-associated determinants. In this study, we performed an integrated phenotypic and genomic analysis of 116 *V. cholerae* isolates collected in 2024 from environmental and clinical sources in Jiaxing, China, including 106 non-O1/non-O139 isolates, 9 O1 isolates, and 1 O139 isolate. Antimicrobial susceptibility testing showed that most isolates remained susceptible to β-lactam/β-lactamase inhibitor combinations, third-generation cephalosporins, carbapenems, and tigecycline, whereas resistance was more frequently observed for ampicillin, streptomycin, nalidixic acid, and ciprofloxacin. Based on the non-susceptibility criteria of Maitrakas et al., 19 of 116 isolates (16.4%) were classified as multidrug-resistant, whereas none met the definition of extensively drug-resistant. Genomic analysis identified diverse resistance determinants, including plasmid-mediated quinolone resistance genes (*qnrVC* variants) and quinolone resistance-determining region mutations in *gyrA* and *parC*. Virulence-associated genes showed heterogeneous distributions: core regulatory and hemolysis-related genes were highly prevalent, whereas classical cholera toxin genes were largely absent. Several accessory virulence factors, including the RTX toxin operon, *chxA*, *ninth*, and *makA*, were detected in subsets of isolates. Core genome multilocus sequence typing revealed substantial genetic diversity, with environmental and clinical isolates distributed across multiple lineages and showing no clear clustering by isolation source. Overall, these data demonstrate the diverse antimicrobial resistance, virulence-associated gene repertoires, and population structure of the Jiaxing *V. cholerae* collection, with particular relevance to the predominant non-O1/non-O139 population.

## 1. Introduction

*Vibrio cholerae* is a widespread aquatic bacterium in freshwater, estuarine, and coastal environments [1,2,3]. While epidemic cholera is caused primarily by toxigenic *V. cholerae* serogroups O1 and O139 [4,5], the majority of environmental *V. cholerae* populations belong to non-O1/non-O139 serogroups (NOVC) [6]. NOVC strains are increasingly detected in natural waters and seafood and are also associated with sporadic human infections, including gastroenteritis, wound infections, and septicemia [7,8,9]. NOVC establish a link between environmental reservoirs and human health, emphasizing their significance in public health monitoring and food safety [10].

Unlike epidemic lineages, NOVC strains generally lack the classical virulence determinants responsible for cholera outbreaks, including cholera toxin (CT, encoded by *ctxA*/*ctxB*) and toxin-coregulated pilus (TCP, encoded by the genes presented in the TCP locus of VPI-1, the key genes included *tcp*-*ABQCRDSTEF*) [11,12]. However, accumulating evidence indicates that NOVC isolates harbor a broad repertoire of alternative virulence-associated genes. These include regulatory and hemolytic factors such as ToxR [13], hemolysinA (HlyA) [13], and hemagglutinin/protease A (HapA) [14]; stress response and enterotoxin-associated factors including RpoS and Stn [13]; as well as cytotoxic factors such as the repeats-in-toxin operon (RTX, *rtxA*/*rtxC*), cholix toxin (ChxA) [15], neuraminidase (NanH) [16], and the membrane-associated cytotoxin MakA [17].

NOVC isolates have also been found to carry variable levels of genes related to colonization and adherence, such as the accessory colonization factor genes (*acfA–acfD*) [13], the mannose-sensitive hemagglutinin pilus (MSHA, *msh* gene cluster) [18], and the outer membrane protein *ompU* [13], together with secretion system-related effectors such as *vasX* and *tagA* [19]. Multiple studies have demonstrated that NOVC virulence potential is shaped by diverse combinations of accessory determinants rather than classical epidemic-associated factors, and that environmental and clinical isolates often share overlapping virulence gene profiles [15,20,21].

The emergence of antimicrobial resistance (AMR) has increased the public health importance of *V. cholerae* [22]. Environmental *V. cholerae* populations are increasingly recognized as reservoirs of AMR genes, influenced by anthropogenic pressures such as wastewater discharge, agricultural activities, and aquaculture [23,24,25,26]. NOVC isolates exhibit diverse antimicrobial susceptibility patterns, including resistance to aminoglycosides, quinolones, tetracyclines, and sulfonamides. Plasmid-mediated quinolone resistance genes, such as *qnrVC* variants, have been frequently detected, along with chromosomal mutations in quinolone resistance-determining regions (QRDR) of DNA gyrase (*gyrA*) and DNA topoisomerase IV (*parC*) [27,28,29,30,31]. The incomplete concordance between genotypic resistance determinants and phenotypic susceptibility reflects the complexity of AMR evolution in *V. cholerae* and emphasizes the value of integrating phenotypic testing with genomic analyses.

Whole-genome sequencing-based phylogenetic analyses reveal high genetic diversity among NOVC isolates, with limited clustering by isolation source, geographic origin, and serogroup [15]. Environmental and clinical isolates were interspersed across the phylogenetic tree, suggesting shared evolutionary backgrounds [15,30]. These phylogenetic patterns are indicative of recombination and environmental adaptation, suggesting an important role of aquatic reservoirs in shaping *V. cholerae* diversity [32].

In this study, we performed an integrated phenotypic and genomic characterization of 116 *V. cholerae* isolates collected from both environmental and clinical sources. By combining antimicrobial susceptibility testing with genomic analyses of resistance determinants, QRDR mutations, virulence-associated gene profiles, and core genome multilocus sequence typing (cgMLST)-based phylogenetic reconstruction, we investigated antimicrobial resistance and pathogenicity diversity, as well as the genetic relatedness of isolates from various origins. Our results provide insight into the genomic landscape of NOVC and inform genomic surveillance strategies targeting environmental reservoirs.

## 2. Materials and Methods

### 2.1. Bacterial Isolates

A total of 116 *V. cholerae* isolates collected in Jiaxing, China, between March and November 2024 were included in this study. These comprised 109 environmental isolates (106 from water samples and 3 from aquatic food products) and 7 clinical isolates (2 from stool samples and 5 from rectal swabs). The isolates originated from multiple districts/counties of Jiaxing, including Haiyan, Pinghu, Haining, Jiashan, and Xiuzhou. Clinical isolates were collected through routine surveillance during the study period. The isolate collection was consecutive, and no preselection was applied prior to phenotypic and genomic analyses. Unless otherwise specified, phenotypic and genomic analyses were conducted on the full set of 116 isolates, including the O1 and O139 isolates, while interpretation focused primarily on the predominant non-O1/non-O139 population. Isolates were grown on lysogeny broth agar (Qingdao Hope Bio-Technology Co., Ltd., Qingdao, China) for 18–24 h at 37 °C before analysis. A single colony was selected and cultured overnight in lysogeny broth at 37 °C [33]. All suspect colonies were identified by oxidase reaction, sodium deoxycholate, and sucrose tests [30]. Only isolates positive for all three tests were selected for further serotyping. These isolates were then serologically examined for agglutination with O1 and O139 antisera (Denka Seiken Co., Ltd., Tokyo, Japan) [23]. This study was approved by the Ethics Committee of the Jiaxing Center for Disease Control and Prevention (Approval number: 2601151453849). All individual data were kept confidential as requested.

### 2.2. Antimicrobial Susceptibility Testing (AST)

The minimum inhibitory concentrations (MICs) of chloramphenicol, co-trimoxazole (trimethoprim–sulfamethoxazole), colistin, ertapenem, meropenem, cefotaxime, ceftazidime, ceftazidime–avibactam, tetracycline, tigecycline, ciprofloxacin, nalidixic acid, azithromycin, streptomycin, ampicillin, and ampicillin–sulbactam were determined using the broth microdilution method. Antimicrobial susceptibility testing was performed using a Sensititre custom broth microdilution plate (Thermo Fisher Scientific, Waltham, MA, USA) according to the manufacturer’s instructions. Bacterial suspensions were prepared to a turbidity equivalent to a 0.5 McFarland standard and inoculated into cation-adjusted Mueller–Hinton broth. Plates were incubated at 35 ± 2 °C for 16–20 h under aerobic conditions. MIC values were interpreted in accordance with the Clinical and Laboratory Standards Institute (CLSI) guidelines (CLSI M100, 2023 edition; M45, 2016 edition). Because no CLSI interpretive criteria are available for colistin against *V. cholerae,* colistin MICs were not categorized as susceptible, intermediate, or resistant and were excluded from MDR/XDR classification. MDR was defined as non-susceptibility, including both intermediate and resistant categories, to at least one agent in three or more antimicrobial categories. XDR was defined as non-susceptibility to at least one agent in all but two or fewer antimicrobial categories, according to the criteria proposed by Magiorakos et al. [34]. For MDR/XDR classification, the tested agents were grouped into the following antimicrobial categories: phenicols, carbapenems, tetracyclines, glycylcyclines, quinolones, antifolates, macrolides, aminoglycosides, penicillins, penicillins plus β-lactamase inhibitors, and third-generation cephalosporins. *Escherichia coli* ATCC 25922, Enterococcus faecalis ATCC 29212, Pseudomonas aeruginosa ATCC 27853, and Staphylococcus aureus ATCC 29213 were used as quality control strains for AST.

### 2.3. Whole-Genome Sequencing and Assembly

Total genomic DNA was extracted from overnight (16–18 h) cultures of all 116 isolates using the QIAamp DNA Mini Kit (Qiagen, Hilden, Germany) following the manufacturer’s instructions. All isolates were subsequently subjected to whole-genome sequencing on the NextSeq 550 platform (Illumina, San Diego, CA, USA) using paired-end reads (2 × 150 bp). Genome assemblies were generated using SPAdes v3.6.0 based on short-read data with default parameters. Assembly quality was assessed using Pathogenwatch assembly statistics, including genome length, number of contigs, N50, and GC content. Across the 116 assemblies, the median genome length was 4,005,012 bp (range, 3,684,864–4,812,932 bp), the median number of contigs was 68 (range, 34–2139), the median N50 was 222,896 bp (range, 2138–636,304 bp), and the median GC content was 47.53% (range, 43.84–47.86%).

### 2.4. Bioinformatic Analysis

AMR genes were identified in all *V. cholerae* genomes using ResFinder (Center for Genomic Epidemiology) with thresholds of ≥95% nucleotide identity and ≥60% coverage [15]. All 116 genomes were screened for QRDR-associated AMR variants in *gyrA* and *parC* using Pathogenwatch [35]. Biotype specific markers and virulence-associated genes were predicted using CholeraFinder with thresholds of ≥95% identity and ≥60% coverage, as previously described [36]. Species identity was further assessed using CholeraFinder 1.0 together with analysis under the *V. cholerae* cgMLST scheme implemented in BacWGSTdb 2.0, comprising 2540 core loci based on the reference strain N16961. Assembled genomes were uploaded to the BacWGSTdb platform, and allelic profiles were generated automatically. A minimum spanning tree (MST) was constructed based on allelic differences to assess the genetic relatedness among isolates [37]. Phylogenetic trees were visualized using Interactive Tree of Life (iTOL) v4 [38].

## 3. Results

### 3.1. V. cholerae Isolate Sources and Serogroup Distribution

**Figure 1:**
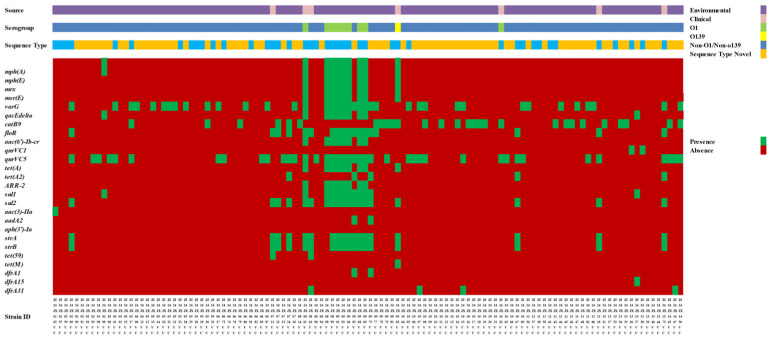
Overview of the distribution of *V. cholerae* isolates included in this study. Sources and serogroup composition of the 116 isolates collected in 2024, including environmental and clinical samples. Environmental isolates were recovered from water and seafood samples, while clinical isolates were obtained from stool samples and rectal swabs. Serogroups (O1, O139, and non-O1/non-O139) are indicated. Light blue means the existed ST type.

A total of 116 *V. cholerae* isolates were included, comprising 109 environmental isolates and 7 clinical isolates (Table 1, Figure 1). Environmental isolates were most commonly collected from water samples (n = 106), with a smaller number recovered from seafood samples (n = 3). Clinical isolates were obtained from stool samples (n = 2) and rectal swabs (n = 5). Of the 116 isolates, 106 belonged to the non-O1/non-O139 group, 9 belonged to serogroup O1, and 1 belonged to serogroup O139.

### 3.2. Antimicrobial Susceptibility, Resistance Genes, and QRDR Mutations

*V. cholerae* isolates exhibited high susceptibility to β-lactam/β-lactamase inhibitor combinations, third-generation cephalosporins, carbapenems, and tigecycline (Table 2, Figure 1). All isolates were susceptible to tigecycline and susceptibility rates > 95% were observed for ampicillin-sulbactam, ceftazidime-avibactam, cefotaxime, ceftazidime, ertapenem, meropenem, amikacin, and co-trimoxazole. Resistance was most frequently observed for ampicillin (33/116, 28.4%), streptomycin (23/116, 19.8%), nalidixic acid (17/116, 14.7%), and ciprofloxacin (13/116, 11.2%), whereas resistance to tetracycline, azithromycin, and chloramphenicol was detected at lower frequencies. Elevated MIC values for colistin were observed in most isolates (114/116, 98.3%); however, no interpretive criteria are available for *V. cholerae*, and therefore no susceptibility categorization was applied. Based on non-susceptibility criteria that included both intermediate and resistant categories, 19 of 116 isolates (16.4%) were classified as MDR, whereas none met the definition of XDR. Colistin was excluded from MDR/XDR classification because no validated interpretive criteria are available for *V. cholerae*. The antimicrobial susceptibility profiles of MDR isolates are summarized in Table S1.

Plasmid-mediated and chromosomally encoded AMR determinants were detected at varying frequencies among the isolates (Table 3). Plasmid-mediated quinolone resistance-associated genes included *qnrVC5* (40/116, 34.4%) and *qnrVC1* (2/116, 1.7%); *aac(6′)-Ib-cr* was identified in 6.8% (8/116) of isolates. Genes associated with macrolide resistance [*mph(A)*, *mph(E)*, *mrx,* and *msr(E)*] were detected in 7.7–8.6% of isolates. Sulfonamide and trimethoprim resistance genes [*sul1* (12/116, 10.3%), *sul2* (19/116, 16.3%), *dfrA1* (2/116, 1.7%), *dfrA15* (1/116, 0.8%), and *dfrA31* (4/116, 3.4%)] were detected at low to moderate frequencies, and the rifampicin resistance gene *ARR-2* was identified in 6.8% of isolates. Chromosomally encoded resistance genes included chloramphenicol resistance determinants [*catB9* (28/116, 24.1%) and *floR* (18/116, 15.5%)], aminoglycoside resistance genes such as *strA* (19/116, 16.3%) and *strB* (19/116, 16.3%), and tetracycline resistance genes [*tet(A)* (9/116, 7.7%), *tet(A2)* (5/116, 4.3%), *tet(59)* (2/116, 1.7%), and *tet(M)* (1/116, 0.8%)]. All 116 isolates were screened for QRDR-associated AMR variants. Relevant QRDR substitutions were identified in 15 isolates, which are summarized in Table 4. The *gyrA* Ser83Ile substitution was detected in all 15 listed isolates, whereas *parC* Ser85Leu was detected in 14 of them; one isolate (ZJ24JX1333VC) lacked the corresponding *parC* substitution. Antimicrobial susceptibility testing against ciprofloxacin and nalidixic acid showed that, among these 15 isolates, 11 were resistant to ciprofloxacin and 11 were resistant to nalidixic acid. The corresponding MIC values and categorical interpretations are presented in Table S2.

### 3.3. Virulence Gene Profiles

Virulence-associated genes were detected at varying frequencies (Table 3). Genes encoding major regulatory and hemolytic factors were highly prevalent, with *toxR* detected in 110 isolates (94.8%), *hlyA* and *hapA* in 113 isolates (97.4%). The enterotoxin gene *stn* was identified in 109 isolates (93.9%), while the stress response regulator *rpoS* was present in 99 isolates (85.3%). The accessory colonization factor genes *acfA*, *acfB*, and *acfC* were identified in 12 (10.3%), 11 (9.4%), and 12 (10.3%) isolates, respectively, whereas *acfD* was detected in 4 isolates (3.4%). The mannose-sensitive hemagglutinin pilus (MSHA) gene cluster was detected in 12 isolates (10.3%), and *ompU* was identified in 4 isolates (3.4%). Genes encoding other virulence-associated factors showed variable distribution. The RTX toxin operon was detected in 38 isolates (32.7%), *nanH* in 37 isolates (31.8%), *chxA* in 26 isolates (22.4%), and *makA* in 20 isolates (17.2%). The *vasX* gene was present in 10 isolates (8.6%) and *tagA* was identified in 11 isolates (9.4%). Additionally, genes associated with quorum sensing and secretion systems, including the Lux operon, were detected in 98 isolates (84.4%). One isolate (ZJ24JX0983VC), identified as serogroup O139, was positive for *ctxA* and *ctxB* but lacked *tcpA* and harbored an incomplete CTX prophage.

### 3.4. Phylogenetic Analysis

**Figure 2:**
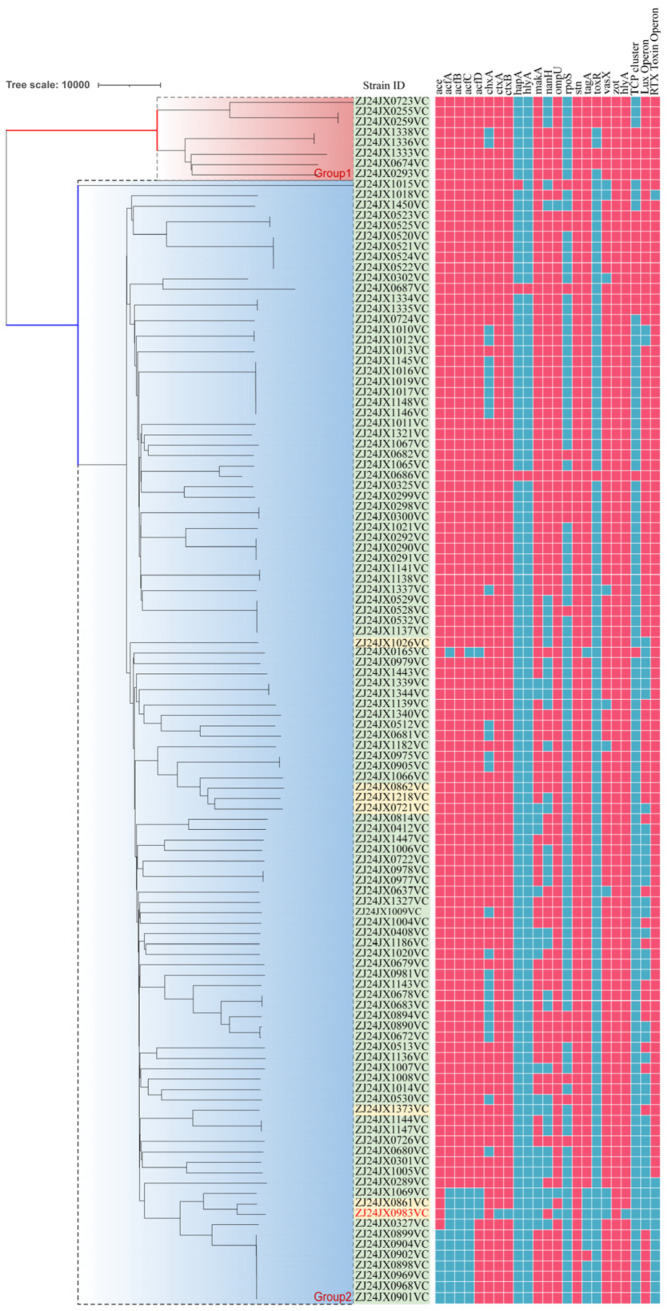
Minimum spanning tree based on core genome multilocus sequence typing (cgMLST) analysis of 116 *V. cholerae* isolates. Assembled genomes were uploaded to the BacWGSTdb database for cgMLST profiling and construction of the minimum spanning tree. Strains are labeled according to isolate ID, with serogroups (O1, O139, and non-O1/non-O139) and isolation sources indicated. Branch lengths represent allelic differences between isolates.

Phylogenetic analysis was performed based on whole-genome sequence data of isolates (Figure 2). The resulting phylogenetic tree showed substantial genetic diversity among the isolates, with strains distributed across multiple distinct lineages. Most isolates belonged to the NOVC serogroup and were scattered throughout the phylogeny, reflecting a heterogeneous genetic background. Clinical and environmental isolates were interspersed across different branches of the tree, with no clear separation based on isolation source. Isolates ZJ24JX0722VC, ZJ24JX0724VC, and ZJ24JX0862VC clustered closely despite being recovered from different sources, while water- and seafood-derived isolates (such as ZJ24JX1218VC and ZJ24JX1333VC) were distributed across distinct lineages that also contained clinical isolates.

The phylogenetic structure was characterized by multiple short internal branches radiating from common nodes, forming a star-like topology. Several small clusters comprising closely related isolates were observed; however, they were limited in size and did not account for the overall population diversity. Isolates ZJ24JX0722VC, ZJ24JX0724VC, and ZJ24JX0862VC were located on neighboring branches with limited allelic differences, whereas isolates such as ZJ24JX1218VC and ZJ24JX1333VC were positioned on more distant branches within the tree. Isolates belonging to the O1 and O139 serogroups were located on distinct branches and did not form a large, closely related cluster. Overall, the phylogenetic analysis revealed a genetically diverse *V. cholerae* population with limited clustering by isolation source or serogroup.

## 4. Discussion

Most *V. cholerae* isolates in this study were susceptible to β-lactam/β-lactamase inhibitor combinations, third-generation cephalosporins, carbapenems, and tigecycline, but were frequently resistant to ampicillin, streptomycin, nalidixic acid, and ciprofloxacin. This profile is consistent with the predominance of environmental NOVC strains in our collection, as previously reported [15,29,39]. Most non-susceptible isolates involved only one or two antimicrobial categories, whereas 19 isolates met the MDR definition. No isolate fulfilled the XDR criteria under the non-susceptibility-based classification framework. Isolate ZJ24JX0721VC showed non-susceptibility to eight antimicrobial categories, while ZJ24JX0165VC showed non-susceptibility to seven categories, and ZJ24JX0290VC and ZJ24JX0299VC each showed non-susceptibility to five categories. This heterogeneity in MDR profiles reflects the genomic coexistence of multiple resistance determinants, highlighting the diverse resistance landscape in environmental NOVC isolates [40,41,42].

A diverse array of AMR-associated genes was detected at the genomic level. Plasmid-mediated quinolone resistance genes, such as *qnrVC5*, were detected at a higher frequency than the phenotypic resistance observed for ciprofloxacin and nalidixic acid. This aligns with previous findings that multiple *qnrVC* alleles, including *qnrVC5*, can be present in *V. cholerae* strains that remain susceptible to ciprofloxacin. Only specific alleles, such as *qnrVC9*, have been associated with increased minimum inhibitory concentrations, suggesting that the effect of *qnrVC* alleles on quinolone susceptibility may vary by allele [31,42,43]. Discrepancies between genotypic determinants and phenotypic susceptibility have previously been observed in *V. cholerae*. The carriage of *qnr* genes is capable of reducing quinolone susceptibility yet may be insufficient for clinical resistance [31,42,43]. Chromosomal mutations within the QRDRs have also been frequently implicated in quinolone resistance in *V. cholerae*. Multiple studies have reported the *gyrA* S83I and *parC* S85L substitutions in clinical and environmental *V. cholerae* isolates, associated with reduced susceptibility to ciprofloxacin and other quinolones [31,33,42]. Similar QRDR mutation patterns were observed in isolates from previous studies, where S83I and S85L mutations co-occurred and were detected alongside plasmid-mediated quinolone resistance genes [42,44,45]; some studies identified *gyrA* mutations at positions S83I, S177A, and S202A, as well as at S85L and I231V in *parC* [33]. Although mutations in the QRDRs such as *gyrA* S83I and *parC* S85L were detected, the isolates (ZJ24JX0722VC, ZJ24JX1333VC) remained phenotypically susceptible to nalidixic acid and ciprofloxacin. Previous studies have shown that *gyrA* S83I and related QRDR mutations can be widespread in seventh pandemic lineages and may not always confer high-level fluoroquinolone resistance in the absence of additional resistance mechanisms [31,44,46]. This discrepancy may reflect incomplete resistance expression, compensatory mutations, low-level resistance below CLSI breakpoints, or possible annotation artifacts in draft genomes.

These findings demonstrate the coexistence of plasmid-mediated and chromosomal quinolone resistance determinants, highlighting the complexity of resistance in NOVC. Resistance genes associated with other antimicrobial classes were detected at low to moderate frequencies, and their presence generally aligned with observed susceptibility profiles [23,47]. The detection of such genes in phenotypically susceptible isolates emphasizes the need to integrate both phenotypic and genomic approaches for comprehensive AMR characterization in *V. cholerae.*

Virulence-associated genes were widely distributed among isolates, although their prevalence varied across functional categories. Core regulatory and hemolytic genes, including *toxR*, *hlyA*, and *hapA* [18,19,48], were detected in the majority of isolates from both environmental and clinical sources, consistent with previous reports of high detection frequencies in NOVC [19,47,49]. Similarly, genes such as *rtxA*/*rtxC* and *hapA* have been reported in isolates from aquatic environments and seafood products [30,50], while classical cholera toxin genes are typically absent in NOVC [10,29,30,51]. The widespread detection of stress response and enterotoxin-associated genes, such as *rpoS* and *stn*, further underscores the potential for diverse virulence repertoires in environmental *V. cholerae* populations [10,48]. By contrast, genes associated with colonization and adherence, such as *acf* and MSHA pilus-related genes, were detected at lower frequencies, reflecting heterogeneous distribution [15,47]. One isolate belonged to serogroup O139 and carried both *ctxA* and *ctxB*. Although toxigenic O139 *V. cholerae* strains have been historically associated with cholera outbreaks [36,52], the isolate identified in this study lacked *tcpA* and harbored an incomplete CTX prophage [52]. As *tcpA* is required for CTXϕ acquisition and intestinal colonization, and an intact CTXϕ is necessary for functional cholera toxin production [36], it is unlikely to be a fully toxigenic or epidemic strain. Its detection in the environment suggests that aquatic environments may serve as reservoirs for toxin-associated genetic fragments rather than classical epidemic *V. cholerae* [47,53]. Several alternative virulence-associated genes were identified in isolate subsets, including those encoding the RTX toxin operon, *chxA*, *nanH*, and *makA*, highlighting the diverse virulence gene repertoire present among NOVC strains [10,47]. Genes encoding the RTX toxin operon (*rtxA*/*rtxC*) were detected in approximately 30–40% of environmental NOVC isolates in recent genomic surveys, consistent with their role in cytotoxicity and host interaction [54]. Similarly, the cholix toxin gene *chxA* and the neuraminidase gene *nanH* were detected at moderate frequencies (~15–25%) in the environmental and food-derived populations. *makA*, encoding a membrane-associated virulence factor, was found in a comparable subset of strains. These observations are consistent with recent reports that NOVC strains frequently carry multiple accessory virulence determinants, even in the absence of classical cholera toxin systems [14,47,54]. Overall, these findings indicate that virulence potential in NOVC appears to be attributable to diverse assortments of accessory virulence-associated genes rather than the presence of classical epidemic determinants.

CgMLST-based phylogenetic analysis revealed a genetically diverse *V. cholerae* population characterized by multiple distinct lineages and a star-like population structure. Clinical and environmental isolates were interspersed throughout without clear clustering by isolation source or serogroup, indicating a heterogeneous population structure. Several small clusters of closely related isolates were identified, although they were limited in size and did not dominate [29,30,47,55,56]. Isolates ZJ24JX0722VC, ZJ24JX0724VC, and ZJ24JX0862VC were located on neighboring branches with limited allelic differences, whereas isolates such as ZJ24JX1218VC and ZJ24JX1333VC were positioned on more distant branches. The distribution of both AMR determinants and virulence-associated genes across multiple phylogenetic backgrounds suggests that these traits are not restricted to a single lineage. Taken together, these results provide a genomic framework for understanding the observed diversity in AMR and virulence gene profiles in *V. cholerae* and highlight the complex population structure of circulating strains in environmental and clinical settings.

This study has several limitations. First, all isolates were collected from a single geographic region, which may limit the generalizability of the observed antimicrobial resistance patterns and population structure. Second, although genomic analyses identified multiple resistance and virulence-associated determinants, functional validation experiments were not performed to confirm their phenotypic effects. In addition, while the sample size was sufficient to reveal substantial genetic diversity, it may not fully represent the broader variability of NOVC populations. Moreover, the relatively small number of clinical isolates precluded formal statistical comparisons between environmental and clinical groups. Broader multicenter sampling and experimental studies will be necessary to help clarify the evolutionary dynamics and pathogenic potential of these strains.

## 5. Conclusions

This study genetically characterized 116 *V. cholerae* isolates from environmental and clinical sources, with particular emphasis on the predominant non-O1/non-O139 population, to investigate antimicrobial resistance profiles, virulence-associated gene content, and phylogenetic relationships. Most isolates remained susceptible to clinically relevant antimicrobials, although resistance to several commonly used agents was observed. Nineteen isolates were classified as multidrug-resistant under a non-susceptibility-based framework, whereas none met the definition of extensively drug-resistant. Accessory virulence-associated genes were identified in isolates from both environmental and clinical origins; however, the limited number of clinical isolates constrained robust inference regarding source-associated patterns. Conventional epidemic determinants were largely absent. Phylogenetic analysis revealed substantial genetic diversity, with no clear clustering according to isolation source or serogroup.

## Figures and Tables

**Table 1:** Source and serogroup distribution of the 116 *V. cholerae* isolates included in this study.

Source	Sample Type	No.
Environment		
	Water	106
	Seafood	3
Clinical		
	Stool	2
	Rectal swab	5
	Total	116
Serogroup	O1	9
	O139	1
	non-O1/non-O139	106
	Total	116

**Table 2:** Antimicrobial susceptibility profiles of the 116 *V. cholerae* isolates from clinical and environmental sources.

Antimicrobial	Antimicrobial Resistance Profile								
	Total (n = 116)			Clinical (n = 7)			Environmental (n = 109)		
	S	I	R	S	I	R	S	I	R
AMP	80 (69.0%)	3 (2.6%)	33 (28.4%)	7 (100.0%)	0 (0.0%)	0 (0.0%)	73 (67.0%)	3 (2.8%)	33 (30.3%)
AMS	113 (97.4%)	0 (0.0%)	3 (2.6%)	7 (100.0%)	0 (0.0%)	0 (0.0%)	106 (97.2%)	0 (0.0%)	3 (2.8%)
CZA	113 (97.4%)	0 (0.0%)	3 (2.6%)	6 (85.7%)	0 (0.0%)	1 (14.3%)	107 (98.2%)	0 (0.0%)	2 (1.8%)
CTX	113 (97.4%)	0 (0.0%)	3 (2.6%)	6 (85.7%)	0 (0.0%)	1 (14.3%)	107 (98.2%)	0 (0.0%)	2 (1.8%)
CTZ	113 (97.4%)	0 (0.0%)	3 (2.6%)	6 (85.7%)	0 (0.0%)	1 (14.3%)	107 (98.2%)	0 (0.0%)	2 (1.8%)
ETP	112 (96.6%)	0 (0.0%)	4 (3.4%)	6 (85.7%)	0 (0.0%)	1 (14.3%)	106 (97.2%)	0 (0.0%)	3 (2.8%)
MEM	113 (97.4%)	0 (0.0%)	3 (2.6%)	6 (85.7%)	0 (0.0%)	1 (14.3%)	107 (98.2%)	0 (0.0%)	2 (1.8%)
CT	Not interpreted	Not interpreted	Not interpreted	Not interpreted	Not interpreted	Not interpreted	Not interpreted	Not interpreted	Not interpreted
AMI	112 (96.6%)	0 (0.0%)	4 (3.4%)	6 (85.7%)	0 (0.0%)	1 (14.3%)	106 (97.2%)	0 (0.0%)	3 (2.8%)
STR	71 (61.2%)	22 (19.0%)	23 (19.8%)	2 (28.6%)	1 (14.3%)	4 (57.1%)	69 (63.3%)	21 (19.3%)	19 (17.4%)
AZM	107 (92.2%)	0 (0.0%)	9 (7.8%)	4 (57.1%)	0 (0.0%)	3 (42.9%)	103 (94.5%)	0 (0.0%)	6 (5.5%)
TET	102 (87.9%)	5 (4.3%)	9 (7.8%)	3 (42.9%)	0 (0.0%)	4 (57.1%)	99 (90.8%)	5 (4.6%)	5 (4.6%)
CIP	101 (87.1%)	2 (1.7%)	13 (11.2%)	4 (57.1%)	0 (0.0%)	3 (42.9%)	97 (89.0%)	2 (1.8%)	10 (9.2%)
NAL	99 (85.3%)	0 (0.0%)	17 (14.7%)	3 (42.9%)	0 (0.0%)	4 (57.1%)	96 (88.1%)	0 (0.0%)	13 (11.9%)
SXT	112 (96.6%)	0 (0.0%)	4 (3.4%)	6 (85.7%)	0 (0.0%)	1 (14.3%)	106 (97.2%)	0 (0.0%)	3 (2.8%)
CHL	108 (93.1%)	5 (4.3%)	3 (2.6%)	6 (85.7%)	0 (0.0%)	1 (14.3%)	102 (93.6%)	5 (4.6%)	2 (1.8%)
TIG	116 (100.0%)	0 (0.0%)	0 (0.0%)	7 (100.0%)	0 (0.0%)	0 (0.0%)	109 (100.0%)	0 (0.0%)	0 (0.0%)

Abbreviations: CHL, chloramphenicol; ETP, ertapenem; MEM, meropenem; TET, tetracycline; TIG, tigecycline; CIP, ciprofloxacin; NAL, nalidixic acid; SXT, co-trimoxazole; AZM, azithromycin; AMI, amikacin; STR, streptomycin; AMP, ampicillin; AMS, ampicillin–sulbactam; CTX, cefotaxime; CTZ, ceftazidime; CZA, ceftazidime–avibactam; CT, colistin. S, susceptible; I, intermediate; R, resistant. Colistin MICs were determined but were not interpreted because no CLSI interpretive criteria are available for *V. cholerae.* Percentages may not total 100.0% because of rounding.

**Table 3:** Prevalence of virulence-associated genes and antimicrobial resistance genes among the 116 *V. cholerae* isolates.

Virulence Genes (%)		Antimicrobial Resistance Genes (%)	
*ace*	7 (6.0%)	*mph(A)*	10 (8.6%)
*acfA*	12 (10.3%)	*mph(E)*	9 (7.7%)
*acfB*	11 (9.4%)	*mrx*	9 (7.7%)
*acfC*	12 (10.3%)	*msr(E)*	9 (7.7%)
*acfD*	4 (3.4%)	*qacEdelta*	10 (8.6%)
*chxA*	26 (22.4%)	*catB9*	28 (24.1%)
*ctxA*	1 (0.8%)	*floR*	18 (15.5%)
*ctxB*	1 (0.8%)	*aac(6′)-Ib-cr*	8 (6.8%)
*hapA*	113 (97.4%)	*qnrVC1*	2 (1.7%)
*hlyA*	113 (97.4%)	*qnrVC5*	40 (34.4%)
*makA*	20 (17.2%)	*tet(A)*	9 (7.7%)
*nanH*	37 (31.8%)	*tet(A2)*	5 (4.3%)
*ompU*	4 (3.4%)	*ARR-2*	8 (6.8%)
*rpoS*	99 (85.3%)	*sul1*	12 (10.3%)
*stn*	109 (93.9%)	*sul2*	19 (16.3%)
*tagA*	11 (9.4%)	*aac(3)-IIa*	1 (0.8%)
*toxR*	110 (94.8%)	*aadA2*	2 (1.7%)
*vasX*	10 (8.6%)	*aph(3′)-Ia*	1 (0.8%)
*zot*	0 (0.0%)	*strA*	19 (16.3%)
*TCP cluster*	1 (0.8%)	*strB*	19 (16.3%)
*Lux Operon*	98 (84.4%)	*tet(59)*	2 (1.7%)
*RTX Toxin Operon*	38 (32.7%)	*tet(M)*	1 (0.8%)
MSHA Pilus	12 (10.3%)	*dfrA1*	2 (1.7%)
		*dfrA15*	1 (0.8%)
		*dfrA31*	4 (3.4%)

**Table 4:** QRDR-associated AMR variant patterns in *gyrA* and *parC* among the 15 isolates in which relevant substitutions were detected by Pathogenwatch. “√” indicates the presence of the indicated substitution, and “/” indicates that the substitution was not detected.

Strain ID	Ciprofloxacin		Nalidixic Acid	
	*gyrA*_S83I	*parC*_S85L	*gyrA*_S83I	*parC*_S85L
ZJ24JX0721VC	√	√	√	√
ZJ24JX0722VC	√	√	√	√
ZJ24JX0724VC	√	√	√	√
ZJ24JX0862VC	√	√	√	√
ZJ24JX0905VC	√	√	√	√
ZJ24JX0975VC	√	√	√	√
ZJ24JX0977VC	√	√	√	√
ZJ24JX0978VC	√	√	√	√
ZJ24JX0979VC	√	√	√	√
ZJ24JX0983VC	√	√	√	√
ZJ24JX1066VC	√	√	√	√
ZJ24JX1067VC	√	√	√	√
ZJ24JX1182VC	√	√	√	√
ZJ24JX1218VC	√	√	√	√
ZJ24JX1333VC	√	/	√	/

## Data Availability

The original contributions presented in this study are included in the article. Further inquiries can be directed to the corresponding authors.

## Supplementary Materials

The following supporting information can be downloaded at: https://www.mdpi.com/article/10.3390/microorganisms14040813/s1, Table S1. Antimicrobial susceptibility profiles of the 19 MDR *Vibrio cholerae* isolates classified according to the non-susceptibility-based criteria of Magiorakos et al. Table S2. Ciprofloxacin and nalidixic acid MIC values and categorical interpretations among the 15 isolates with QRDR-associated substitutions.
