# Supplementary material for: Integrated Phenotypic and Genomic Analysis of Antimicrobial Resistance, Virulence, and Phylogeny in Vibrio cholerae Isolates from Jiaxing, China, with Emphasis on Non-O1/Non-O139 Strains
Source: Microorganisms. 2026 Apr 2;14(4):813. doi: 10.3390/microorganisms14040813 (PMC13118530; doi:10.3390/microorganisms14040813)
Supplement: Supplementary file 1 [file microorganisms-14-00813-s001.zip › microorganisms-4182402-supplementary.pdf]

Table S1. Antimicrobial susceptibility profiles of the 19 MDR *Vibrio cholerae* isolates classified according to the non-susceptibility-based criteria of Magiorakos et al.

Strain ID	CHL	ETP	MEM	TET	TIG	CIP	NAL	SXT	AZM	AMI	STR	AMP	AMS	CTX	CTZ	CZA	Number of non-susceptible antimicrobial categories
ZJ24JX0721VC	R	R	R	R	S	R	R	S	R	R	R	S	S	R	R	R	8
ZJ24JX0165VC	I	S	S	I	S	I	S	R	S	S	R	R	R	S	S	S	7
ZJ24JX0290VC	S	R	S	S	S	I	R	S	S	S	I	R	S	R	R	R	5
ZJ24JX0299VC	S	R	R	S	S	R	R	S	S	S	I	R	S	R	R	R	5
ZJ24JX0327VC	S	S	S	S	S	S	R	S	S	S	R	R	R	S	S	S	4
ZJ24JX0898VC	I	S	S	R	S	S	S	S	S	S	R	S	S	S	S	S	4
ZJ24JX0899VC	I	S	S	I	S	S	S	S	S	S	R	S	S	S	S	S	4
ZJ24JX0901VC	R	S	S	R	S	S	S	S	S	S	R	S	S	S	S	S	4
ZJ24JX0902VC	I	S	S	R	S	S	S	S	S	S	R	S	S	S	S	S	4
ZJ24JX0968VC	R	S	S	I	S	S	S	S	S	S	R	S	S	S	S	S	4
ZJ24JX0969VC	I	S	S	R	S	S	S	S	S	S	R	S	S	S	S	S	4
ZJ24JX0975VC	S	S	S	I	S	S	R	S	S	S	R	S	S	S	S	S	4
ZJ24JX0983VC	S	S	S	R	S	R	R	S	R	S	R	S	S	S	S	S	4
ZJ24JX0674VC	S	S	S	S	S	S	S	S	S	S	R	R	R	S	S	S	3
ZJ24JX0724VC	S	S	S	S	S	R	R	S	S	S	R	S	S	S	S	S	3
ZJ24JX0861VC	S	S	S	R	S	S	S	S	S	S	R	S	S	S	S	S	3
ZJ24JX0862VC	S	S	S	R	S	R	R	S	S	S	R	S	S	S	S	S	3
ZJ24JX0904VC	S	S	S	I	S	R	R	S	S	S	R	S	S	S	S	S	3
ZJ24JX0905VC	S	S	S	R	S	R	R	S	S	S	R	S	S	S	S	S	3

Abbreviations: CHL, chloramphenicol; ETP, ertapenem; MEM, meropenem; TET, tetracycline; TIG, tigecycline; CIP, ciprofloxacin; NAL, nalidixic acid; SXT, co-trimoxazole; AZM, azithromycin; AMI, amikacin; STR, streptomycin; AMP, ampicillin; AMS, ampicillin–sulbactam; CTX, cefotaxime; CTZ, ceftazidime; CZA, ceftazidime–avibactam.

S, susceptible; I, intermediate; R, resistant.

MDR was defined as non-susceptibility to at least one agent in three or more antimicrobial categories according to Magiorakos et al. For MDR/XDR classification, the tested

agents were grouped into the following antimicrobial categories: phenicols, carbapenems, tetracyclines, glycylcyclines, quinolones, antifolates, macrolides, aminoglycosides, penicillins, penicillins plus β-lactamase inhibitors, and third-generation cephalosporins. Colistin was excluded because no CLSI interpretive criteria are available for *Vibrio cholerae*.

Table S2. Ciprofloxacin and nalidixic acid MIC values and categorical interpretations among the 15 isolates with QRDR-associated substitutions.

Strain ID	CIP MIC (mg/L)	CIP category	NAL MIC (mg/L)	NAL category
ZJ24JX0721VC	2	R	>32	R
ZJ24JX0722VC	0.24	R	8	S
ZJ24JX0724VC	1	R	>32	R
ZJ24JX0862VC	>2	R	>32	R
ZJ24JX0905VC	2	R	>32	R
ZJ24JX0975VC	0.06	S	32	R
ZJ24JX0977VC	2	R	>32	R
ZJ24JX0978VC	2	R	>32	R
ZJ24JX0979VC	2	R	>32	R
ZJ24JX0983VC	1	R	>32	R
ZJ24JX1066VC	0.24	R	<4	S
ZJ24JX1067VC	2	R	<4	S
ZJ24JX1182VC	<0.015	S	>32	R
ZJ24JX1218VC	<0.015	S	>32	R
ZJ24JX1333VC	<0.015	S	<4	S

Abbreviations: CIP, ciprofloxacin; NAL, nalidixic acid; MIC, minimum inhibitory concentration.
